# Co-occurrence of habit-forming risk behaviors and their socio-demographic, health status and lifestyle determinants: a population-based cross-sectional study

**DOI:** 10.1186/s13690-024-01251-2

**Published:** 2024-02-28

**Authors:** Junko Kose, Pauline Duquenne, Serge Hercberg, Pilar Galan, Mathilde Touvier, Léopold K. Fezeu, Valentina A. Andreeva

**Affiliations:** 1 Nutritional Epidemiology Research Group (EREN), Epidemiology and Statistics Research Center, Sorbonne Paris Nord University, INSERM U1153, INRAE U1125, CNAM, University of Paris (CRESS), Bobigny, France; 2 Department of Public Health, AP-HP Paris Seine-Saint-Denis Hospital System, Bobigny, France

**Keywords:** Addictive behaviors, Alcohol, Food addiction, Internet addiction, Substance use disorders, General population

## Abstract

**Background:**

Although habit-forming risk behaviors frequently co-occur, determinants of concurrent risk behaviors have rarely been investigated. The aim of the present study was to investigate socio-demographic, health status, and lifestyle determinants of single versus concurrent risk behaviors in general-population adults.

**Methods:**

We analyzed data from 32,622 participants (74.5% female; mean age = 57.9 ± 14.2 years) of the NutriNet-Santé cohort who completed the Alcohol Use Disorders Identification Test, the 12-item Cigarette Dependence Scale, the modified Yale Food Addiction Scale 2.0, and the Internet Addiction Test in 2021–2022. Using established cutoffs, participants were first split into 2 groups (presence versus absence) for each risk variable (alcohol use disorders, nicotine dependence, food addiction, Internet addiction) and were then divided into 3 groups (no risk behavior, 1 risk behavior (reference), and ≥ 2 risk behaviors). The association between socio-demographic, health status, and lifestyle exposures and individual/concurrent risk behaviors were investigated with polytomous logistic regression.

**Results:**

Younger age (Odds Ratio (OR) = 2.04; 95% Confidence Interval (CI: 1.62–2.56), current financial difficulties (OR = 1.29; CI: 1.08–1.54), self-perceived poor health (OR = 1.70; CI: 1.32–2.20), overall poor dietary quality (OR = 2.88; CI: 2.06–4.02), being underweight (OR = 1.46; CI: 1.05–2.04), having obesity (OR = 1.62; CI: 1.31–1.99), lack of affection during childhood (OR = 1.41; CI: 1.18–1.69), and a lifetime prevalence or medication use for a mental disorder (OR = 1.46; CI: 1.24–1.73) were positively associated with having ≥ 2 versus 1 risk behavior (all *p* < 0.05). The comparison of none versus 1 risk behavior revealed the same determinants in addition to having a higher education, being physically active at work, and being overweight.

**Conclusions:**

We investigated determinants of concurrent habit-forming risk behaviors among adults in a large, population-based study. The findings could serve as impetus for future research in this domain and ultimately help guide addiction prevention efforts.

**Supplementary Information:**

The online version contains supplementary material available at 10.1186/s13690-024-01251-2.

**Table:** 

**Text box 1. Contributions to the literature**
• Concurrent mental health disorders are generally associated with increased severity and poorer prognosis.
• Younger age, financial difficulties, poor self-perceived health and diet quality, not being of normal weight, lack of affection during childhood, and a lifetime prevalence of other mental disorders were determinants of concurrent versus individual habit-forming/addictive behaviors in this large population-based study.
• This study helps to identify vulnerable population subgroups to be targeted by addiction prevention programs.

## Introduction

Substance use disorders (SUD) are underscored by a loss of control as well as seeking or compulsive use of substances despite negative aftereffects [1]. Among the ten substances addressed in the Diagnostic and Statistical Manual of Mental Disorders – 5th edition (DSM-5), alcohol and tobacco are the most widely used substances around the world, with serious health consequences [2, 3]. The most recent World Health Organization estimates indicate that 43% of the global population aged ≥ 15 years are current drinkers [4] and 22.3% use tobacco [3]. Among French adults, current data show that 10% reported to be daily drinkers [5] and 30.4% reported to be smokers [6]. As regards alcohol use disorders (AUD), the average lifetime prevalence was estimated at 8.6% according to the World Mental Health Survey including studies conducted between 2001 and 2015 in 27 countries around the world [7]. In turn, the concept of food addiction (FA) was first introduced in the 1950s [8] yet remains controversial to this day [9]. Research interest in FA has spiked in recent years, given the popularity of industrially processed, highly palatable, and intensely marketed foods [10, 11]. A meta-analysis including 272 studies from around the world estimated a pooled prevalence of FA at 14% in non-clinical samples [12]. The DSM-5 also defines non-substance-related disorders [1], which are increasingly attracting research attention. Internet gaming disorder – while not yet recognized as an addiction – is mentioned in the DSM-5 as meriting further research [1]. According to a meta-analysis published in 2022 and including 341 studies, the global prevalence of Internet addiction (IA) in all ages was estimated at 14.2% [13]. It is also important to note the prevalent comorbidity, i.e., the presence or accumulation of ≥ 2 distinct health conditions in the same individual [14], of these habit-forming risk behaviors. Indeed, numerous studies have been conducted on the co-occurrence of AUD–nicotine dependence (ND) [15]. Comorbidity is generally associated with increased symptom severity of each disorder and with a poorer prognosis [14].

Considering the heavy burden of these habit-forming risk behaviors, it is crucial to explore associated socio-demographic, health status, and lifestyle determinants in order to inform addiction prevention efforts. Thus far, epidemiological studies have suggested determinants of individual risk behavior. The most prominent determinants include: younger age [16–19], male sex [13, 20, 21], adverse childhood experiences [20], low socio-economic status [17, 20], low education [18, 20], experiencing a stressful life event [20], being unmarried [17], low physical activity [22, 23], not being of normal weight [19, 24, 25], having a low healthy lifestyle score (with 9 dimensions including nutrition and exercise) [26], and having other mental health conditions [23, 24, 27, 28]. However, limitations of the existing studies include the use of specific homogeneous samples, such as young adults, medical students, and smokers [20–22, 24], and studying each risk behavior separately. Even though studies suggest a high correlation among these behaviors [15, 29–34], there is a paucity of research on concurrent habit-forming risk behaviors and associated factors. Therefore, the aim of the present study was to describe socio-demographic, health status, and lifestyle determinants of individual and concurrent habit-forming risk behaviors in a sample recruited from the general population. A greater focus was placed on identifying determinants of the number rather than the type of habit-forming risk behaviors. We use the term “habit-forming risk behavior” rather than “addictive behavior” because the assessment (described below) was based on self-reported information, not on clinical diagnoses of addiction, and also because some of the behaviors are not yet officially recognized as addictive. Over the last few years, habit formation theory has gained in prominence for explaining addiction [35] which underscores difficulty in overcoming habitual responses [36]. We hypothesized that individuals with known risk factors would be more likely to exhibit concurrent rather than a single risk behavior.

## Methods

### The NutriNet-Santé cohort

NutriNet-Santé is an ongoing prospective cohort launched in France in 2009. The main objectives are to investigate the multifaceted relationship between nutrition and health, as well as the determinants of dietary behaviors and physical activity. Its design and protocol were detailed elsewhere [37]. Briefly, recurrent multimedia calls target adults aged 18 years and older who are able to follow an Internet-based study protocol in French (https://etude-nutrinet-sante.fr/). NutriNet-Santé was approved by the Institutional Review Board of the French Institute for Health and Medical Research and by the National Commission on Informatics and Liberty. The cohort is registered at: https://clinicaltrials.gov/ct2/show/NCT03335644. Eligible volunteers provide informed consent prior to enrollment.

At inclusion and every year thereafter, participants are asked to complete a set of questionnaires regarding socio-demographic and lifestyle characteristics, anthropometrics, physical activity, diet (every six months), and health status. Additional nutrition- or health-related questionnaires (described below) are sent to participants on a regular basis as part of the follow-up.

### Data collection

#### Main outcomes: habit-forming risk behavior assessment

In NutriNet-Santé, risk behavior assessment took place between July 2021 and January 2022 using a comprehensive questionnaire. Of the 151,397 participants who received it, a total of 33,985 returned the questionnaire within 6 months and were thus eligible for this analysis. The present study deals with four types of risk behaviors: AUD, ND, FA, and IA. Each type of risk behavior was first dichotomized (presence or absence, as detailed below) and then participants were split into three groups: no risk behavior, 1 risk behavior (any type), and ≥ 2 risk behaviors.

#### Alcohol Use Disorders Identification Test (AUDIT)

The AUDIT was developed by a six-country World Health Organization collaborative project as a screening instrument for harmful alcohol consumption and alcohol dependence [38]. This questionnaire contains 10 items: 3 items for alcohol consumption (frequency, amount, and heavy use); 5 items for drinking behavior over the past year, which are scored on a 5-point Likert scale ranging from 0 to 4 points; and 2 items for alcohol-related problems (i.e., injuries due to drinking, concern by a friend or doctor for one’s drinking) scored with 0, 2, or 4 points each. The total score is thus 40 points and a score ≥ 8 is considered an indicator of hazardous and harmful alcohol use [38]. Compared with a DSM-5- defined moderate to severe AUD, an 8-point cut-off presented sensitivity and specificity of 73% and 90%, respectively, in males, 76% and 97%, respectively, in females [39]. The French version of AUDIT demonstrated high internal consistency (Cronbach’s alpha = 0.87) in a sample of 1,207 adults (mean age = 43.2 ± 17.2 years; 51.6% females) [40].

#### 12-Item Cigarette Dependence Scale (CDS-12)

The CDS-12 was originally developed and validated in the French language on the basis of qualitative and quantitative data on cigarette dependence in an international Internet-based sample of 3,009 smokers (age range 18–70 years) [41]. This 12-item questionnaire covers the key aspects of the definitions of dependence from the DSM-IV and the International Classification of Diseases 10th Revision, namely compulsion, withdrawal symptoms, loss of control, time allocation, neglect of other activities, and persistence despite harm [41]. Each item is scored on a 5-point Likert scale ranging from 1 to 5 points with a maximum score of 60 points [41]. The CDS-12 showed a high test-retest reliability (*r* = 0.84), and a high internal consistency (Cronbach’s alpha = 0.90) in the original sample [41]. The cut-off of 43 points showed the highest sensitivity (64%) and specificity (68%) when compared against ND assessed by the Mini International Neuropsychiatric Interview [42]. As the CDS-12 has been validated against ND as defined in the DSM-IV, this terminology was retained in the present study, even when citing studies using other terms (e.g., tobacco use disorders).

#### Modified Yale Food Addiction Scale 2.0 (mYFAS 2.0)

The mYFAS 2.0 was derived from the YFAS 2.0 including 35 items of the DSM-5 criteria for substance-related and addictive disorders applied to highly palatable food [43]. The mYFAS 2.0 consists of 13 items: one item for each of the 11 DSM-5 diagnostic criteria and 2 items for impairment and distress experienced over the past year [43]. Each item was first scored on an 8-point Likert scale ranging from 0 (never) to 7 (every day). Then, item-specific cut-off values [43] were applied to dichotomize each item (presence versus absence of risk). Participants with ≥ 1 positive response (i.e., presence) on impairment or distress and ≥ 2 positive responses on any of the 11 items assessing DSM-5 diagnostic criteria were considered as having a FA phenotype [43]. The French version of the mYFAS 2.0 displayed significant correlations with the YFAS 2.0 (*r* = 0.83), Body Mass Index (BMI) (*r* = 0.17), binge eating (*r* = 0.42), cognitive restraint (*r* = 0.24), uncontrolled eating (*r* = 0.30), and emotional eating (*r* = 0.31) in a non-clinical sample (*n* = 250; mean age = 28.4 ± 11.3 years; 80.0% females) [43].

#### Internet Addiction Test (IAT)

The IAT is a validated and widely used questionnaire regarding problematic Internet use [44]. It contains 20 items assessing symptoms of IA: user’s preoccupation with the Internet, ability to control use, extent of hiding or lying about one’s Internet use, and continued Internet use despite negative consequences [45]. Each item is scored on a 5-point Likert scale ranging from 1 (never) to 5 (always) points with a maximum score of 100 points [45]. A score of ≥ 50 points identifies problematic Internet use [45]. The French version of IAT showed good psychometric properties in a sample of 246 participants (mean age = 24.1 ± 9.0 years, range 18–54 years; 67.0% females): Cronbach’s alpha = 0.93; significant correlations were reported with daily duration of Internet use (*r* = 0.53) and age (*r*=-0.23) [45].

#### Main exposures: socio-demographic, health status, and lifestyle characteristics

Established determinants of individual habit-forming risk behavior were selected as exposure variables in the present study. A validated socio-demographic questionnaire was used to collect self-reported data on age which was modeled as categories (18–39; 40–59; ≥60 years), sex (female; male), and educational level (less than high school; high school diploma or equivalent; some college, undergraduate degree; graduate degree) [46]. The online version of this questionnaire was validated in comparison with a standard paper-and-pencil questionnaire [46]. As regards height and weight, a validated self-reported anthropometric questionnaire was used [47], which allowed us to calculate BMI (kg/m^2^). Participants were split into four categories according to their anthropometric status (underweight: <18.5, normal weight: 18.5–24.9, overweight: 25.0–29.9, and obese: ≥30.0 kg/m^2^). The validity of the self-reported height and weight online was established in comparison with clinical measurements and with a paper-and-pencil version of the tool [47]. As the socio-demographic and anthropometric questionnaires are administered at baseline and annually thereafter, data recorded on the date closest to the assessment date of the risk behaviors were used for the present study. Data on self-reported lifetime prevalence and/or medication use for other mental health conditions (yes; no) were collected by an annual health status questionnaire.

Information on current household financial situation (comfortable or good; barely making it; in debt) [48], self-perceived health status (very good or good; acceptable; poor or very poor) [48], self-perceived dietary quality (excellent or very good; good or acceptable; poor) [49], smoking status (never smoker; former smoker; current smoker) [46], e-cigarette use (yes; no) [48], current alcohol use (0 glass/week; <2 glasses/week; ≤2–6 glasses/week; ≥7 glasses/week), self-perceived lack of affection during childhood (yes; no), type of profession (mostly active; mostly sedentary; retired or other), and prior divorce (yes; no) was collected at the same time as the habit-forming risk behaviors assessment. It should be noted that smoking (regular and e-cigarettes) and drinking were retained as potential determinants since they do not by themselves refer to problematic substance use. The latter is defined not only by the frequency or amount of consumption, but also by other important criteria, such as tolerance, withdrawal, compulsion, loss of control, chronicity of the behavior, and deleterious consequences in daily life [1].

### Statistical analyses

Participants with incomplete data on risk behaviors were excluded from this analysis. Likewise, participants lacking data on BMI in a 5-year window around the risk behavior assessment and those lacking data on educational level were also excluded. The remaining covariables did not have any missing values.

Descriptive characteristics are presented in the full sample and according to sex, given evidence for associations between sex and each habit-forming risk behavior [13, 20, 21]. The data reflect number (percent) from chi-squared tests (categorical variables) and mean (± SD) from Student’s *t*-test (continuous variables). For the main analyses, the associations between socio-demographic, health status, and lifestyle characteristics (exposure variables) and habit-forming risk behavior status (outcome variable categorized as: no risk behavior, 1 risk behavior = reference, and ≥ 2 risk behaviors) were assessed using polytomous logistic regression. In a sensitivity analysis, the same associations were assessed using no risk behavior as reference. Since we were interested in investigating sex as a determinant of the number of risk behaviors, the main analyses were conducted in the full sample. The following exposure variables were modelled in the main analysis and were mutually adjusted for: sex, age category, education, type of profession, prior divorce, current household financial situation, self-perceived dietary quality, BMI category, tobacco smoking status, current e-cigarette use, current alcohol use, lack of affection during childhood, and self-reported lifetime prevalence or medication use for a mental health condition. All tests were two-sided and *p* < 0.05 was considered as evidence for statistical significance. SAS version 9.4 (SAS Institute, Inc., Cary NC, USA) was used for all statistical analyses.

## Results

### Description of study sample

**Fig. 1:**
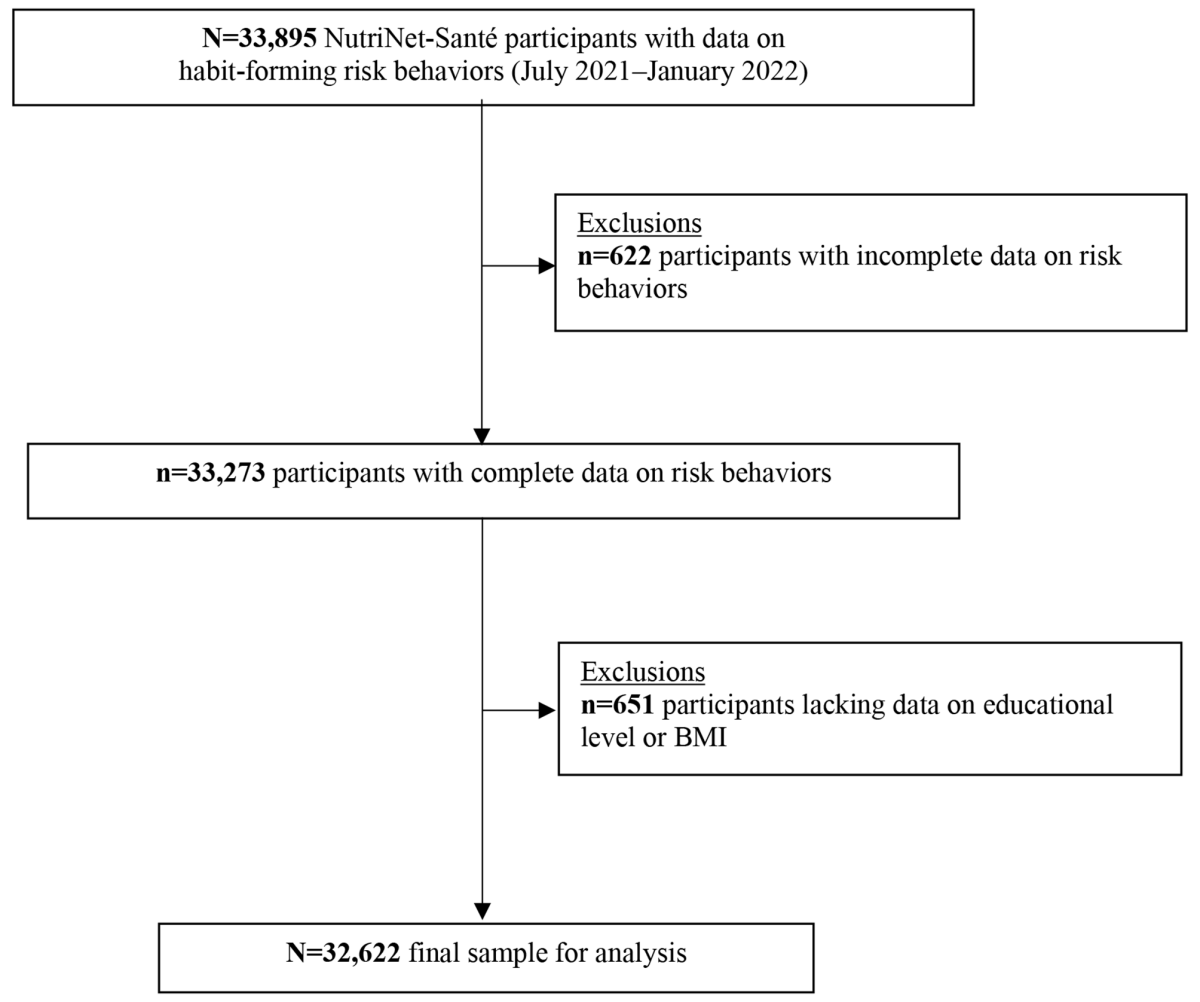
Participant selection flowchart

In total, 33,273 participants had complete data on habit-forming risk behaviors. After excluding those lacking data on BMI or educational level, the final sample for the analysis included 32,622 adults [participant flowchart presented in Fig. 1] (74.5% female; mean age = 57.9 ± 14.2 years). Participants included in the analysis were older, more likely to be male, retired, former smokers, to have good self-perceived health status and good dietary quality, less likely to have AUD, IA, or to experience financial difficulties compared to those excluded from the analysis (data not tabulated). The grouping of participants according to habit-forming risk behavior status was as follows: “no risk behavior” *n* = 27,036 (82.9%); “1 risk behavior, any type” *n* = 4,702 (14.4%); “≥2 risk behaviors” *n* = 884 (2.7%).

Table 1 presents the participants’ socio-demographic and lifestyle characteristics in the full sample and according to sex. Female participants were more likely to be younger, underweight or normal weight, to experience current financial difficulties, to have an undergraduate degree, self-perceived poor dietary quality, were less likely to be retired, former smokers, and to consume alcohol (in terms of frequency and/or quantity) compared to male participants (all *p* < 0.0001).

**Table 1:** Descriptive characteristics of the participants in the full sample and according to sex (*N* = 32,622; NutriNet-Santé cohort; 2021–2022; France)

	Full sample *N* = 32,622		Males *n* = 8,307		Females *n* = 24,315		*p*-value^a^
**Age, years, mean (SD)**	57.9	(14.2)	62.6	(13.6)	56.3	(14.0)	< 0.001
**Age category**							
18–39 years	4,305	(13.2)	618	(7.4)	3,687	(15.2)	< 0.001
40–59 years	11,560	(35.4)	2,360	(28.4)	9,200	(37.8)	
≥ 60 years	16,757	(51.4)	5,329	(64.2)	11,428	(47.0)	
**Educational level**							
Less than high school	4,024	(12.3)	1,221	(14.7)	2,803	(11.5)	< 0.001
High school diploma or equivalent	5,033	(15.4)	1,347	(16.2)	3,686	(15.2)	
Some college, undergraduate	10,197	(31.3)	2,122	(25.5)	8,075	(33.2)	
Graduate degree	13,368	(41.0)	3,617	(43.5)	9,751	(40.1)	
**Type of professional activity**							
Mostly sedentary	12,874	(39.5)	2,702	(32.5)	10,172	(41.8)	< 0.001
Mostly active	4,520	(13.9)	865	(10.4)	3,655	(15.0)	
Retired	14,574	(44.7)	4,685	(56.4)	9,889	(40.7)	
Other^b^	654	(2.0)	55	(0.7)	599	(2.5)	
**Prior divorce**							
No	24,704	(75.7)	6,343	(76.4)	18,361	(75.5)	0.12
Yes	7,918	(24.3)	1,964	(23.6)	5,954	(24.5)	
**Current household financial situation**							
Comfortable, good	27,612	(84.6)	7,243	(87.2)	20,369	(83.8)	< 0.001
Barely making it	4,862	(14.9)	1,031	(12.4)	3,831	(15.8)	
In debt	148	(0.5)	33	(0.4)	115	(0.5)	
**Self-perceived health status**							
Very good, good	22,094	(67.7)	5,614	(67.6)	16,480	(67.8)	0.81
Acceptable	8,925	(27.4)	2,274	(27.4)	6,651	(27.4)	
Poor or very poor	1,603	(4.9)	419	(5.0)	1,184	(4.9)	
**Self-perceived dietary quality**							
Excellent, very good	13,013	(39.9)	3,608	(43.4)	9,405	(38.7)	< 0.001
Good, acceptable	19,171	(58.8)	4,615	(55.6)	14,556	(59.9)	
Poor	438	(1.3)	84	(1.0)	354	(1.5)	
**Body mass index (BMI; kg/m** ^**2**^ **), mean (SD)**	24.3	(4.7)	25.1	(3.9)	24.0	(4.9)	< 0.001
**BMI category**							
Underweight (< 18.5)	1,614	(5.0)	101	(1.2)	1,513	(6.2)	< 0.001
Normal weight (18.5–24.9)	19,289	(59.1)	4,482	(54.0)	14,807	(60.9)	
Overweight (25.0–29.9)	8,197	(25.1)	2,864	(34.5)	5,333	(21.9)	
Obesity (≥ 30.0)	3,522	(10.8)	860	(10.4)	2,662	(11.0)	
**Tobacco smoking status**							
Never smoker	21,243	(65.1)	4,847	(58.4)	16,396	(67.4)	< 0.001
Former smoker	8,841	(27.1)	2,911	(35.0)	5,930	(24.4)	
Current smoker	2,538	(7.8)	549	(6.6)	1,989	(8.2)	
**Current e-cigarette use**							
No	31,831	(97.6)	8,130	(97.9)	23,701	(97.5)	0.09
Yes, without nicotine	128	(0.4)	25	(0.3)	103	(0.4)	
Yes, with nicotine	663	(2.0)	152	(1.8)	511	(2.1)	
**Alcohol use, # glasses/week** ^**c**^							
0	4,735	(14.5)	769	(9.3)	3,966	(16.3)	< 0.001
< 2	15,198	(46.6)	3,035	(36.5)	12,163	(50.0)	
2–6	11,275	(34.6)	3,747	(45.1)	7,528	(31.0)	
≥ 7	1,414	(4.3)	756	(9.1)	658	(2.7)	

Values refer to number (%) except when noted otherwise

^a^ Values are obtained from Chi-2 or Student t-tests, as appropriate

^b^ Without professional activity (homemaker, sick leave, unemployment, parental leave, disability) or not specified

^c^ 1 glass = 10 g of ethanol

Table 2 presents participants’ psychological and mental health characteristics in the full sample and according to sex. Compared to males, females were more likely to report a lack of affection during childhood, to have ND, FA, other mental health conditions, and were less likely to have AUD (all *p* < 0.0001).

**Table 2:** Psychological and mental health characteristics of the participants in the full sample and according to sex (*N* = 32,622; NutriNet-Santé cohort; 2021–2022; France)

	Full sample *N* = 32,622		Males *n* = 8,307		Females *n* = 24,315		*p*-value^a^
**Lack of affection during childhood**							
No	28,409	(87.1)	7,627	(91.8)	20,782	(85.5)	< 0.001
Yes	4,213	(12.9)	680	(8.2)	3,533	(14.5)	
**Self-reported lifetime prevalence or medication use for a mental health condition** ^**b**^							
No	18,359	(56.3)	5,738	(69.1)	12,621	(51.9)	< 0.001
Yes	14,263	(43.7)	2,569	(30.9)	11,694	(48.1)	
**Alcohol use disorders** ^**c**^							
No	30,289	(92.8)	7,349	(88.5)	22,940	(94.3)	< 0.001
Yes	2,333	(7.2)	958	(11.5)	1,375	(5.7)	
**Nicotine dependence** ^**c**^							
No	32,043	(98.2)	8,211	(98.8)	23,832	(98.0)	< 0.001
Yes	579	(1.8)	96	(1.2)	483	(2.0)	
**Food addiction** ^**c**^							
No	30,958	(94.9)	8,155	(98.2)	22,803	(93.8)	< 0.001
Yes	1,664	(5.1)	152	(1.8)	1,512	(6.2)	
**Internet addiction** ^**c**^							
No	30,627	(93.9)	7,806	(94.0)	22,821	(93.9)	0.71
Yes	1,995	(6.1)	501	(6.0)	1,494	(6.1)	
**Number of habit-forming risk behaviors** ^**c**^							
0	27,036	(82.9)	6,833	(82.3)	20,203	(83.1)	0.08
1	4,702	(14.4)	1,266	(15.2)	3,436	(14.1)	
2	788	(2.4)	185	(2.2)	603	(2.5)	
3	91	(0.3)	21	(0.3)	70	(0.3)	
4	5	(0.0)	2	(0.0)	3	(0.0)	

Values refer to number (%)

^a^ Values are obtained from Chi-2 tests

^b^ Mental health conditions include memory impairment, Alzheimer’s disease, anorexia nervosa, anxiety disorders, bipolar disorder, depression, and insomnia

^c^ Habit-forming risk behaviors include alcohol use disorders, nicotine dependence, food addiction, and Internet addiction, which were assessed by the Alcohol Use Disorders Identification Test (≥ 8 points), the 12-item Cigarette Dependence Scale (≥ 43 points), the modified Yale Food Addiction Scale 2.0, and the Internet Addiction Test (≥ 50 points), respectively. Scoring for each scale was based on established criteria

The frequency distributions of the participants having a combination of two risk behaviors (*n* = 788), and those having a combination of three risk behaviors (*n* = 91) are presented in Supplementary tables S1 and S2, respectively.

### Associations between socio-demographic, health status, and lifestyle characteristics and habit-forming risk behavior status

Table 3 shows the main results obtained from adjusted polytomous logistic regression in the full sample. Younger age, current financial difficulties, self-perceived poor health and poor dietary quality, being underweight, having obesity, current tobacco use, heavy alcohol use, lack of affection during childhood, and a lifetime prevalence or medication use for a mental health condition were positively associated with having ≥ 2 risk behaviors compared to having 1 risk behavior (reference) (all *p* < 0.05). The significant ORs ranged from 1.29 (CI: 1.08–1.54) for current financial difficulties to 2.88 (CI: 2.06–4.02) for self-perceived poor dietary quality. Non-significant results were observed for sex (OR = 0.89; CI: 0.73–1.07), high educational attainment (OR = 0.99; CI: 0.76–1.28), not being physically active at work (OR = 1.10; CI 0.88–1.37), a prior divorce (OR = 0.87; CI: 0.72–1.05), and current e-cigarette use (OR = 1.09; CI: 0.82–1.44).

**Table 3:** Associations of socio-demographic, health status, and lifestyle characteristics with number of habit-forming risk behaviors^a^ (*N* = 32,622, reference = 1 risk behavior; NutriNet-Santé cohort; 2021–2022; France)

	No risk behavior *n* = 27,036			≥ 2 risk behaviors *n* = 884			Overall *p*-value^b^
	OR^b^	(95% CI)^b^	*p*-value^b^	OR^b^	(95% CI)^b^	*p*-value^b^	
**Sex**							0.125
Female	1			1			
Male	0.92	(0.85–1.00)	0.06	0.89	(0.73–1.07)	0.21	
**Age category**							< 0.001
18–39 years	0.20	(0.18–0.22)	< 0.001	2.04	(1.62–2.56)	< 0.001	
40–59 years	0.46	(0.42–0.50)	< 0.001	1.32	(1.09–1.60)	0.004	
≥ 60 years	1			1			
**Educational level**							< 0.001
Less than high school	1			1			
High school diploma or equivalent	0.90	(0.78–1.04)	0.15	0.99	(0.73–1.34)	0.94	
Some college, undergraduate, graduate degree	0.77	(0.68–0.86)	< 0.001	0.99	(0.76–1.28)	0.91	
**Type of professional activity**							< 0.001
Mostly sedentary, retired, other^c^	0.83	(0.75–0.91)	< 0.001	1.10	(0.88–1.37)	0.39	
Mostly active	1			1			
**Prior divorce**							0.148
No	1			1			
Yes	0.93	(0.86–1.01)	0.10	0.87	(0.72–1.05)	0.14	
**Current household financial situation**							< 0.001
Comfortable, good	1			1			
Barely making it, in debt	0.85	(0.78 − 0.94)	< 0.001	1.29	(1.08–1.54)	0.005	
**Self-perceived health status**							< 0.001
Very good, good	1			1			
Acceptable	0.76	(0.70–0.82)	< 0.001	1.30	(1.09–1.55)	0.003	
Poor or very poor	0.53	(0.46–0.61)	< 0.001	1.70	(1.32–2.20)	< 0.001	
**Self-perceived dietary quality**							< 0.001
Excellent, very good	1			1			
Good, acceptable	0.63	(0.58–0.68)	< 0.001	1.39	(1.14–1.69)	< 0.001	
Poor	0.20	(0.16–0.26)	< 0.001	2.88	(2.06–4.02)	< 0.001	
**BMI (kg/m** ^**2**^ **) category**							< 0.001
Underweight (< 18.5)	0.95	(0.81–1.12)	0.55	1.46	(1.05–2.04)	0.03	
Normal weight (18.5–24.9)	1			1			
Overweight (25.0–29.9)	0.76	(0.70–0.83)	< 0.001	1.18	(0.98–1.43)	0.08	
Obesity (≥ 30.0)	0.54	(0.48–0.60)	< 0.001	1.62	(1.31–1.99)	< 0.001	
**Tobacco smoking status**							< 0.001
Never smoker	1			1			
Former smoker	0.74	(0.68–0.80)	< 0.001	0.96	(0.80–1.16)	0.69	
Current smoker	0.33	(0.29–0.36)	< 0.001	1.97	(1.62–2.39)	< 0.001	
**Current e-cigarette use**							< 0.001
No	1			1			
Yes	0.71	(0.59–0.85)	< 0.001	1.09	(0.82–1.44)	0.56	
**Alcohol use, # glasses/week** ^**d**^							< 0.001
0	1			1			
< 2	1.07	(0.96–1.20)	0.19	1.05	(0.80–1.37)	0.74	
2–6	0.63	(0.56–0.70)	< 0.001	1.86	(1.43–2.43)	< 0.001	
≥ 7	0.05	(0.04–0.06)	< 0.001	2.82	(2.10–3.80)	< 0.001	
**Lack of affection during childhood**							< 0.001
No	1			1			
Yes	0.58	(0.53–0.63)	< 0.001	1.41	(1.18–1.69)	< 0.001	
**Self-reported lifetime prevalence or medication use for a mental health condition** ^**e**^							
No	1			1			< 0.001
Yes	0.63	(0.58–0.67)	< 0.001	1.46	(1.24–1.73)	< 0.001	

BMI: Body Mass Index; CI: Confidence Interval; OR: Odds Ratio

^a^ Habit-forming risk behaviors include alcohol use disorders, nicotine dependence, food addiction, and Internet addiction, which were assessed by the Alcohol Use Disorders Identification Test (≥ 8 points), the 12-item Cigarette Dependence Scale (≥ 43 points), the modified Yale Food Addiction Scale 2.0, and the Internet Addiction Test (≥ 50 points), respectively

^b^ Values are obtained from a polytomous logistic regression model (reference = 1 risk behavior; *n* = 4,702). Variables are mutually adjusted

^c^ Other = Without professional activity (homemaker, sick leave, unemployment, parental leave, disability) or not specified

^d^ 1 glass = 10 g of ethanol

^e^ Mental health conditions include memory impairment, Alzheimer’s disease, anorexia nervosa, anxiety disorders, bipolar disorder, depression, and insomnia

All significant determinants of having ≥ 2 risk behaviors, plus having a higher educational attainment, being physically active at work, being overweight, and current e-cigarette use were inversely associated with having none versus 1 risk behavior (reference). The significant ORs ranged from 0.05 (CI: 0.04–0.06) for heavy alcohol use to 0.85 (CI: 0.78–0.94) for current financial difficulties (all *p* < 0.05). Results for sex (OR = 0.92; CI: 0.85–1.00) and a prior divorce (OR = 0.93; CI: 0.86–1.01) were not significant.

Results of the sensitivity analysis, modelling no risk behavior as reference, are presented in Supplementary table S3. We observed significant results for the same determinants as those seen in the main analysis and linear trends with higher odds as the number of risk behaviors increased.

## Discussion

This large population-based study revealed that a number of socio-demographic, health status, and lifestyle characteristics were associated with having none, a single or concurrent (≥ 2) habit-forming risk behaviors, which supported our main hypothesis. When comparing ≥ 2 versus 1 habit-forming risk behavior, significantly increased ORs were observed for younger age, current household financial difficulties, self-perceived poor health and poor dietary quality, being underweight, having obesity, current tobacco use, current heavy alcohol use, lack of affection during childhood, and a lifetime prevalence or medication use for a mental health condition. In turn, high educational level, not being physically active at work, and current e-cigarette use were significantly associated with having a single (but not concurrent) risk behavior versus having no risk behaviors. Interestingly, sex and prior divorce did not emerge as significant determinants of either single or concurrent risk behaviors.

Consistent with our findings, previous studies have linked socio-demographic, health status, and lifestyle characteristics with individual risk behaviors. Specifically, younger age was shown to be a determinant of SUD [50], FA [18, 19], and IA [16, 17], respectively; adverse childhood experiences were shown to be a determinant of SUD [20]; poor dietary quality or disordered eating have been associated with alcohol use [51], FA [52, 53], and IA [54, 55], respectively; not being of normal weight has been associated with FA [19, 24, 56] and IA [25], respectively; smoking has been associated with AUD [29], FA [31, 32], and IA [33], respectively; alcohol use has been associated with ND [29] and IA [34]; finally, presence of other mental health conditions has been associated with SUD [50], FA [23, 24, 27, 56], and IA [28], respectively. The clustering of determinants has been evoked by descriptive studies showing a positive association of occupational sitting time with education and income [57]. In the addiction literature, educational level was positively associated with SUD [20] and inversely associated with IA [13]. People with a high educational level might be more likely to consume alcohol than those with a lower educational level [58]; moreover, having a higher educational attainment has been positively associated with heavy episodic drinking in young adults [58]. Such findings could partially explain our results. Next, low physical activity and sedentariness have been positively associated with hazardous drinking [59]. Prior studies have also linked low physical activity with IA and FA, respectively [22, 23]. Future studies are needed to shed more light on the role of sedentariness and physical activity in concurrent habit-forming risk behaviors.

Previous research has suggested the co-occurrence of each pair of AUD, ND, FA, and IA [29, 34, 54]. For example, a review reported the co-existence of SUD and IA [34]; a cross-sectional study including 36,309 adults revealed a significant association between AUD and ND [29]. In a systematic review and meta-analysis including cross-sectional and prospective studies, eating underscored by a loss of control and binge eating disorders, which are highly correlated with FA [56, 60, 61], were reported to be associated with IA [54]. A cross-sectional study also suggested significant associations between problematic Internet use and eating disorders in both males and females [62]. The preoccupation with the Internet, in particular, was a strong predictor of eating disorders in that study [62]. In terms of the underlying mechanisms, SUD and other habit-forming risk behaviors might share the same neurobiological basis (e.g., dopamine reward system), genetic overlap, and psychosocial antecedents (e.g., impulsivity) [63]. Studies have also reported neurobiological and psychological parallels between ND or SUD and FA, such as activation of the dopaminergic reward pathways, opioid and cannabinoid systems, gut-brain axis mechanisms, chronic stress during childhood affecting the nervous, endocrine, and immune systems, and emotional development [32, 50, 56, 64]. Moreover, a literature review suggested that co-occurrence of SUD and IA could be explained by shared psychosocial factors, such as low self-esteem, poor family functioning, low life satisfaction, and personality more likely to engage in a behavior perceived as rewarding [34]. In turn, postulated shared neurobiological mechanisms between alcohol and tobacco use include cross-reinforcement and cross-tolerance [15]. The former refers to each substance’s role in increasing consumption of the other substance by impacting the mesolimbic dopamine pathway and the latter indicates that nicotine reduces the sedative and intoxication effects of alcohol, leading to increased alcohol use [15]. Given that comorbidity has been linked to increased symptom severity and poorer prognosis of each individual disorder [14], our results, along with future studies in this domain, could help identify the most at-risk populations to be targeted by prevention efforts aiming to improve lifestyle behaviors and reduce the likelihood of habits leading to addiction.

In the present study, we were interested in investigating alcohol consumption, tobacco smoking status, and e-cigarette use as determinants of the number rather than the type of habit-forming risk behaviors. This decision was driven by the fact that problematic substance use takes into account not only the frequency or amount of consumption, but also tolerance, withdrawal, compulsion, loss of control, chronicity of the behavior, and deleterious consequences in daily life [1]. A study based on general-population surveys from 9 European countries reported a weak correlation between frequency of alcohol use and the AUDIT total score [65]. In addition, the majority of participants with excessive drinking in repeated cross-sectional studies of U.S. representative samples [66] did not meet the alcohol dependence criteria. Indeed, in the present study, the Spearman’s correlation coefficient between alcohol use (glasses/week) and AUDIT score was modest (*r* = 0.35; *p* < 0.001). In prior studies, binge drinking and alcohol dependence were shown to have distinct determinants; the prevalence of alcohol dependence was estimated at 10.5% among binge drinkers and 1.3% among non-binge drinkers [66]. As regards tobacco use, a longitudinal study with a representative U.S. sample reported that nearly half of smokers did not have ND as defined by the DSM-IV [67]. Indeed, individual differences in nicotine reinforcement and withdrawal according to sex, anthropometric characteristics, and mental health status have been evoked [68], which could help explain differences in ND among smokers. In addition, addiction to a substance is strongly linked to the age at initiation [69]. Finally, e-cigarettes might hold a reduced addictive potential than conventional cigarettes [70]. In our study, current e-cigarette use did not emerge as a significant determinant of concurrent risk behaviors, possibly owing to the relatively small number of participants reporting this behavior.

Limitations of the present study must be noted. First, even though the risk behaviors were estimated with instruments validated against or based on DSM diagnostic criteria, which argues for the utility of self-reported measures in epidemiological research [40, 42, 43, 45], they do not correspond to clinical diagnoses of addiction. Moreover, FA and IA are not defined in the DSM-5 [1]. Second, the administration of the exposure questionnaire (July 2021–January 2022) coincided with the COVID-19 pandemic, albeit not at its onset. This unintended aspect might have impacted to some extent the assessed prevalence of the risk behaviors [13, 71]. However, a French national survey has reported stable alcohol and tobacco consumption levels during and outside the lockdown periods [72]. Thus, the results are likely not subject to a strong bias owing to the pandemic which, nonetheless, should be taken into consideration when extrapolating the results. Third, the main results are presented in the full sample because we hypothesized that sex might be a determinant of the number of habit-forming risk behaviors [13, 20, 21]. Fourth, in the present study, the ancillary protocol called for the assessment of ND only among current smokers, thus it was not possible to provide a correlation between smoking status and ND. Fifth, this was a descriptive study using a cross-sectional design; as such, it cannot provide a basis for examining the direction of the associations. Importantly, reverse causation cannot be ruled out. Sixth, caution is needed when generalizing the findings because individuals with SUD are generally less likely to participate in epidemiological research than their SUD-free counterparts [73], which may lead to under-estimation of the studied associations. Also, as seen in epidemiological research in general, the NutriNet-Santé cohort includes a higher proportion of females, individuals with higher educational and socio-economic levels, and a lower smoking prevalence compared to the French general population [74]. This aspect could lead to underestimation of observed associations and should also be taken into account when extrapolating the findings. Future studies could investigate the prevalence of habit-forming risk behaviors in representative samples of the general population and in specific subgroups in order to establish moderators of the associations. Likewise, future mediation analyses could provide evidence for causal pathways.

Despite the limitations, this study has several important strengths. To our knowledge, this is the first large-scale epidemiological study to reveal factors associated with individual versus concurrent habit-forming risk behaviors in a heterogenous sample of adults recruited from the general population. To date, most of the research in this domain has focused on adolescents and young adults [20–22, 24]. Our study, where the mean age was 57.9 years, could help advance knowledge about determinants of habit-forming risk behaviors in middle age. In addition, we were interested in identifying determinants of multiple concurrent habit-forming risk behaviors, hence we used one risk behavior as reference in the main analysis.

In conclusion, the present study suggests some socio-demographic, health status, and lifestyle determinants of concurrent habit-forming risk behaviors. Future longitudinal studies could elucidate the direction and causality of the observed associations. The present and future findings could help identify targets for addiction prevention efforts at the population level in order to reduce the likelihood of deleterious habits turning into addiction.

### Electronic supplementary material

Below is the link to the electronic supplementary material.

Supplementary Material 1

## Data Availability

Researchers at public institutions can submit a project collaboration request that includes information about their institution and a brief description of the project to: collaboration@etude-nutrinet-sante.fr. All requests are reviewed by the steering committee of the NutriNet-Santé study. In case of approval, a signed data access agreement will be requested and additional authorizations from the competent administrative authorities may be needed regarding human subjects’ data protection. In accordance with existing regulations, no personally identifiable data will be made available.

## Abbreviations

AUD: Alcohol use disorders
AUDIT: Alcohol Use Disorders Identification Test
BMI: Body Mass Index
CI: Confidence interval
CDS-12: 12-item Cigarette Dependence Scale
DSM: Diagnostic and Statistical Manual of Mental Disorders
FA: Food addiction
IA: Internet addiction
IAT: Internet Addiction Test
ND: Nicotine dependence
mYFAS: modified Yale Food Addiction Scale
OR: Odds ratio
SUD: Substance use disorders

## References

[1] American Psychiatric Association (2013). Diagnostic and Statistical Manual of Mental Disorders. 5th Ed (DSM-5).

[2] World Health Organization. Alcohol [Internet]. 2022 [cited 2023 Dec 27]. Available from: https://www.who.int/news-room/fact-sheets/detail/alcohol.

[3] World Health Organization. Tobacco [Internet]. 2022 [cited 2023 Dec 27]. Available from: https://www.who.int/news-room/fact-sheets/detail/tobacco.

[4] World Health Organization. Global status report on alcohol and health 2018. Geneva: WHO; 2018.

[5] Richard J, Andler R, Cogordan C, Spilka S, Nguyen-Thanh V. La consommation d’alcool chez les adultes en France en 2017 [Alcohol consumption among adults in France in 2017]. Bull Epidémiol Hebd. 2019;5–6:89–97.

[6] Pasquereau A (2020). Consommation de tabac parmi les adultes: bilan de cinq années de programme national contre le tabagisme, 2014–2019 [Tobacco use among adults: five-year review of the national tobacco control programme, 2014–2019]. Bull Epidémiol Hebd.

[7] Glantz MD, Bharat C, Degenhardt L, Sampson NA, Scott KM, Lim CC (2020). The epidemiology of alcohol use disorders cross-nationally: findings from the World Mental Health Surveys. Addict Behav.

[8] Randolph TG (1956). The descriptive features of food addiction; addictive eating and drinking. Q J Stud Alcohol.

[9] Gearhardt AN, Hebebrand J (2021). The concept of food addiction helps inform the understanding of overeating and obesity: debate consensus. Am J Clin Nutr.

[10] Gearhardt AN, Schulte EM (2021). Is food addictive? A review of the science. Annu Rev Nutr.

[11] Monteiro CA, Cannon G, Lawrence M, Costa Louzada M, Pereira Machado P (2019). Ultra-processed foods, diet quality, and health using the NOVA classification system.

[12] Praxedes DRS, Silva-Júnior AE, Macena ML, Oliveira AD, Cardoso KS, Nunes LO (2022). Prevalence of food addiction determined by the Yale Food Addiction Scale and associated factors: a systematic review with meta-analysis. Eur Eat Disord Rev.

[13] Meng SQ, Cheng JL, Li YY, Yang XQ, Zheng JW, Chang XW (2022). Global prevalence of digital addiction in general population: a systematic review and meta-analysis. Clin Psychol Rev.

[14] Valderas JM, Starfield B, Sibbald B, Salisbury C, Roland M (2009). Defining comorbidity: implications for understanding health and health services. Ann Fam Med.

[15] Adams S (2017). Psychopharmacology of tobacco and alcohol comorbidity: a review of current evidence. Curr Addict Rep.

[16] Tsumura H, Kanda H, Sugaya N, Tsuboi S, Takahashi K (2018). Prevalence and risk factors of internet addiction among employed adults in Japan. J Epidemiol.

[17] Sela Y, Bar-Or RL, Kor A, Lev-Ran S. The Internet Addiction Test: psychometric properties, socio-demographic risk factors and addictive co-morbidities in a large adult sample. Addict Behav. 2021;122:107023. 10.1016/j.addbeh.2021.107023 34198053

[18] Kircaburun K, Ünübol H, Sayar GH, Stavropoulos V, Griffiths MD. Measurement, prevalence, and psychological risk factors associated with addictive food consumption: development of a new food addiction scale and evidence from a national large-scale sample. J Behav Addict. 2020;9(3):836–52. 10.1556/jba-9-836 PMC8943666 32903203

[19] Schulte EM, Gearhardt AN (2018). Associations of food addiction in a sample recruited to be nationally representative of the United States. Eur Eat Disord Rev.

[20] Stone AL, Becker LG, Huber AM, Catalano RF (2012). Review of risk and protective factors of substance use and problem use in emerging adulthood. Addict Behav.

[21] Choi SW, Kim DJ, Choi JS, Ahn H, Choi EJ, Song WY (2015). Comparison of risk and protective factors associated with smartphone addiction and internet addiction. J Behav Addict.

[22] Derbyshire KL, Lust KA, Schreiber LRN, Odlaug BL, Christenson GA, Golden DJ (2013). Problematic internet use and associated risks in a college sample. Compr Psychiatry.

[23] Li JTE, Pursey KM, Duncan MJ, Burrows T (2018). Addictive eating and its relation to physical activity and sleep behavior. Nutrients.

[24] Romero-Blanco C, Hernández-Martínez A, Parra-Fernández ML, Onieva-Zafra MD, Prado-Laguna M, del C, Rodríguez-Almagro J (2021). Food addiction and lifestyle habits among university students. Nutrients.

[25] Aghasi M, Matinfar A, Golzarand M, Salari-Moghaddam A, Ebrahimpour-Koujan S (2020). Internet use in relation to overweight and obesity: a systematic review and meta-analysis of cross-sectional studies. Adv Nutr.

[26] Kim CH, Kang KA, Shin S (2022). Healthy lifestyle status related to alcohol and food addiction risk among college students: a logistic regression analysis. J Am Coll Health.

[27] Burrows T, Kay-Lambkin F, Pursey K, Skinner J, Dayas C (2018). Food addiction and associations with mental health symptoms: a systematic review with meta-analysis. J Hum Nutr Diet.

[28] Andrade ALM, Scatena A, Bedendo A, Enumo SRF, Dellazzana-Zanon LL, Prebianchi HB (2020). Findings on the relationship between internet addiction and psychological symptoms in Brazilian adults. Int J Psychol.

[29] Chou SP, Goldstein RB, Smith SM, Huang B, Ruan WJ, Zhang H (2016). The epidemiology of DSM-5 nicotine use disorder: results from the national epidemiologic survey on alcohol and related conditions-III. J Clin Psychiatry.

[30] Hoover LV, Yu HP, Cummings JR, Ferguson SG, Gearhardt AN. Co-occurrence of food addiction, obesity, problematic substance use, and parental history of problematic alcohol use. Psychol Addict Behav. 2023;37(7):928–35. 10.1037/adb0000870 PMC10986778 35878078

[31] Owari Y, Miyatake N, Suzuki H. Relationship between food dependence and nicotine dependence in smokers: a cross-sectional study of staff and students at medical colleges. Medicina (Kaunas). 2019;55(5):202. 10.3390/medicina55050202 PMC6571764 31126155

[32] Zawertailo L, Attwells S, deRuiter WK, Le TL, Dawson D, Selby P (2020). Food addiction and tobacco use disorder: common liability and shared mechanisms. Nutrients.

[33] Wang J, Hao Q hong, Tu Y, Peng W, Wang Y, Li H et al. Assessing the association between internet addiction disorder and health risk behaviors among adolescents and young adults: a systematic review and meta-analysis. Front Public Health. 2022;10:809232. 10.3389/fpubh.2022.809232 PMC9010676 35433568

[34] Ko CH, Yen JY, Yen CF, Chen CS, Chen CC (2012). The association between internet addiction and psychiatric disorder: a review of the literature. Eur Psychiatry.

[35] Sjoerds Z, Luigjes J, van den Brink W, Denys D, Yücel M (2014). The role of habits and motivation in human drug addiction: a reflection. Front Psychiatry.

[36] McKim TH, Bauer DJ, Boettiger CA (2016). Addiction history associates with the propensity to form habits. J Cogn Neurosci.

[37] Hercberg S, Castetbon K, Czernichow S, Malon A, Mejean C, Kesse E (2010). The NutriNet-Santé study: a web-based prospective study on the relationship between nutrition and health and determinants of dietary patterns and nutritional status. BMC Public Health.

[38] Babor TF, Higgins-Biddle JC, Saunders JB, Monteiro MG. Alcohol Use Disorders Identificaiton Test: guidelines for use in primary care. 2nd ed. Geneva: World Health Organization; 2001.

[39] Moehring A, Rumpf HJ, Hapke U, Bischof G, John U, Meyer C. Diagnostic performance of the Alcohol Use Disorders Identification Test (AUDIT) in detecting DSM-5 alcohol use disorders in the general population. Drug Alcohol Depend. 2019;204:107530. 10.1016/j.drugalcdep.2019.06.032 31505375

[40] Gache P, Michaud P, Landry U, Accietto C, Arfaoui S, Wenger O, et al. The Alcohol Use Disorders Identification Test (AUDIT) as a screening tool for excessive drinking in primary care: reliability and validity of a French version. Alcohol Clin Exp Res. 2005;29(11):2001–7. 10.1097/01.alc.0000187034.58955.64 16340457

[41] Etter JF, Le Houezec J, Perneger TV. A self-administered questionnaire to measure dependence on cigarettes: The Cigarette Dependence Scale. Neuropsychopharmacology. 2003;28(2):359–70. 10.1038/sj.npp.1300030 12589389

[42] Etter JF. Comparing the validity of The Cigarette Dependence Scale and the Fagerström Test for nicotine dependence. Drug Alcohol Depend. 2008;95(1–2):152–9. 10.1016/j.drugalcdep.2008.01.017 18328641

[43] Brunault P, Berthoz S, Gearhardt AN, Gierski F, Kaladjian A, Bertin E (2020). The modified Yale Food Addiction Scale 2.0: validation among non-clinical and clinical french-speaking samples and comparison with the full Yale Food Addiction Scale 2.0. Front Psychiatry.

[44] Laconi S, Rodgers RF, Chabrol H (2014). The measurement of internet addiction: a critical review of existing scales and their psychometric properties. Comput Hum Behav.

[45] Khazaal Y, Billieux J, Thorens G, Khan R, Louati Y, Scarlatti E, et al. French validation of the Internet Addiction Test. Cyberpsychol Behav. 2008;11(6):703–6. 10.1089/cpb.2007.0249 18954279

[46] Vergnaud AC, Touvier M, Méjean C, Kesse-Guyot E, Pollet C, Malon A (2011). Agreement between web-based and paper versions of a socio-demographic questionnaire in the NutriNet-Santé study. Int J Public Health.

[47] Lassale C, Péneau S, Touvier M, Julia C, Galan P, Hercberg S (2013). Validity of web-based self-reported weight and height: results of the NutriNet-Santé study. J Med Internet Res.

[48] Santé publique France. Baromètre santé. Saint-Maurice: SPF; 2017

[49] Loftfield E, Yi S, Immerwahr S, Eisenhower D (2015). Construct validity of a single-item, self-rated question of diet quality. J Nutr Educ Behav.

[50] Benowitz NL (2010). Nicotine addiction. N Engl J Med.

[51] Cummings JR, Gearhardt AN, Ray LA, Choi AK, Tomiyama AJ (2020). Experimental and observational studies on alcohol use and dietary intake: a systematic review. Obes Rev.

[52] Pursey KM, Collins CE, Stanwell P, Burrows TL (2015). Foods and dietary profiles associated with ‘food addiction’ in young adults. Addict Behav Rep.

[53] Burrows T, Hides L, Brown R, Dayas CV, Kay-Lambkin F (2017). Differences in dietary preferences, personality and mental health in Australian adults with and without food addiction. Nutrients.

[54] Hinojo-Lucena FJ, Aznar-Díaz I, Cáceres-Reche MP, Trujillo-Torres JM, Romero-Rodríguez JM. Problematic internet use as a predictor of eating disorders in students: a systematic review and meta-analysis study. Nutrients. 2019;11(9):2151. 10.3390/nu11092151 PMC6769899 31505749

[55] Ioannidis K, Taylor C, Holt L, Brown K, Lochner C, Fineberg NA (2021). Problematic usage of the internet and eating disorder and related psychopathology: a multifaceted, systematic review and meta-analysis. Neurosci Biobehav Rev.

[56] Florio L, Lassi DLS, de Azevedo-Marques Perico C, Vignoli NG, Torales J, Ventriglio A (2022). Food addiction: a comprehensive review. J Nerv Ment Dis.

[57] De Cocker K, Duncan MJ, Short C, van Uffelen JGZ, Vandelanotte C (2014). Understanding occupational sitting: prevalence, correlates and moderating effects in Australian employees. Prev Med.

[58] Lui CK, Kerr WC, Mulia N, Ye Y (2018). Educational differences in alcohol consumption and heavy drinking: an age-period-cohort perspective. Drug Alcohol Depend.

[59] Hallgren M, Vancampfort D, Nguyen TTD, Ekblom-Bak E, Wallin P, Andersson G (2021). Physical activity, sedentary behavior, and cardiorespiratory fitness in hazardous and non-hazardous alcohol consumers. Am J Health Promot.

[60] Hauck C, Cook B, Ellrott T (2020). Food addiction, eating addiction and eating disorders. Proc Nutr Soc.

[61] di Giacomo E, Aliberti F, Pescatore F, Santorelli M, Pessina R, Placenti V (2022). Disentangling binge eating disorder and food addiction: a systematic review and meta-analysis. Eat Weight Disord.

[62] Yildirim MS (2018). Investigation of the relationship between risk of internet addiction, food addiction, and self-esteem in high school students. Dusunen Adam J Psychiatry Neurol Sci.

[63] Shaffer HJ, LaPlante DA, LaBrie RA, Kidman RC, Donato AN, Stanton MV (2004). Toward a syndrome model of addiction: multiple expressions, common etiology. Harv Rev Psychiatry.

[64] Seoane-Collazo P, Diéguez C, Nogueiras R, Rahmouni K, Fernández-Real JM, López M (2021). Nicotine’ actions on energy balance: friend or foe?. Pharmacol Ther.

[65] Knibbe RA, Derickx M, Kuntsche S, Grittner U, Bloomfield K (2006). A comparison of the alcohol use disorder identification test (AUDIT) in general population surveys in nine European countries. Alcohol Alcohol.

[66] Esser MB, Hedden SL, Kanny D, Brewer RD, Gfroerer JC, Naimi TS (2014). Prevalence of alcohol dependence among US adult drinkers, 2009–2011. Prev Chronic Dis.

[67] Grant BF, Shmulewitz D, Compton WM (2020). Nicotine use and DSM-IV nicotine dependence in the United States, 2001–2002 and 2012–2013. Am J Psychiatry.

[68] U.S. Department of Health and Human Services (2010). How tobacco smoke causes disease: the biology and behavioral basis for smoking-attributable disease: a report of the surgeon general.

[69] American Cancer Society. Why people start using tobacco, and why it’s hard to stop [Internet]. [cited 2023 Dec 27]. Available from: https://www.cancer.org/healthy/stay-away-from-tobacco/why-people-start-using-tobacco.html.

[70] Shiffman S, Sembower MA (2020). Dependence on e-cigarettes and cigarettes in a cross‐sectional study of US adults. Addiction.

[71] Zvolensky MJ, Garey L, Rogers AH, Schmidt NB, Vujanovic AA, Storch EA (2020). Psychological, addictive, and health behavior implications of the COVID-19 pandemic. Behav Res Ther.

[72] Santé publique France. CoviPrev: une enquête pour suivre l’évolution des comportements et de la santé mentale pendant l’épidémie de COVID-19 [CoviPrev: a survey to track changes in behavior and mental health during the COVID-19 epidemic] [Internet]. [cited 2023 Dec 27]. Available from: https://www.santepubliquefrance.fr/etudes-et-enquetes/coviprev-une-enquête-pour-suivre-l’évolution-des-comportements-et-de-la-sante-mentale-pendant-l-epidemie-de-covid-19.

[73] Bu F (2022). Non-response and attrition in longitudinal studies. J Epidemiol Community Health.

[74] Andreeva VA, Salanave B, Castetbon K, Deschamps V, Vernay M, Kesse-Guyot E (2015). Comparison of the sociodemographic characteristics of the large NutriNet-Santé e-cohort with French census data: the issue of volunteer bias revisited. J Epidemiol Community Health.

