# Supplementary material for: Co-occurrence of habit-forming risk behaviors and their socio-demographic, health status and lifestyle determinants: a population-based cross-sectional study
Source: Arch Public Health. 2024 Feb 28;82:26. doi: 10.1186/s13690-024-01251-2 (PMC10900606; doi:10.1186/s13690-024-01251-2)
Supplement: Supplementary file 1 — Supplementary Material 1 [file 13690_2024_1251_MOESM1_ESM.docx]

**Supplementary table S1**. Frequency distribution of each pair of risk behaviors (n=788; NutriNet-Santé cohort; 2021-2022; France)

| Risk behavior pairs | n | % |
| --- | --- | --- |
| Food + Internet | 272 | 34.5 |
| Alcohol + Internet | 222 | 28.2 |
| Alcohol + Food | 132 | 16.8 |
| Alcohol + Tobacco | 111 | 14.1 |
| Tobacco + Internet | 28 | 3.6 |
| Tobacco + Food | 23 | 2.9 |
| Total | 788 | 100.0 |

**Supplementary table S2**. Frequency distribution of each combination of three risk behaviors (n=91; NutriNet-Santé cohort; 2021-2022; France)

| Combinations of 3 risk bejaviors | n | % |
| --- | --- | --- |
| Alcohol + Food + Internet | 62 | 68.1 |
| Alcohol + Tobacco + Internet | 12 | 13.2 |
| Tobacco + Food + Internet | 10 | 11.0 |
| Alcohol + Tobacco + Food | 7 | 7.7 |
| Total | 91 | 100.0 |

**Supplementary table S3**. Sensitivity analysis : Associations of socio-demographic, health status, and lifestyle characteristics with number of habit-forming risk behaviors^a^ (N=32,622; reference=no risk behavior; NutriNet-Santé cohort; 2021-2022; France)

|  | 1 risk behavior  n=4,702 | | | ≥ 2 risk behaviors  n=884 | | | Overall  *p*-value^b^ |
| --- | --- | --- | --- | --- | --- | --- | --- |
|  | OR^b^ | (95% CI)^b^ | *p*-value^b^ | OR^b^ | (95% CI)^b^ | *p*-value^b^ |  |
| **Sex** |  |  |  |  |  |  | 0.12 |
| Female | 1 |  |  | 1 |  |  |  |
| Male | 1.08 | (1.00–1.18) | 0.06 | 0.96 | (0.78–1.16) | 0.68 |  |
| **Age category** |  |  |  |  |  |  | <0.001 |
| 18-39 years | 2.17 | (2.00–2.36) | <0.001 | 2.87 | (2.38–3.46) | <0.001 |  |
| 40-59 years | 5.09 | (4.57–5.66) | <0.001 | 10.37 | (8.27–13.00) | <0.001 |  |
| ≥ 60 years | 1 |  |  | 1 |  |  |  |
| **Educational level** |  |  |  |  |  |  | <0.001 |
| Less than high school | 1 |  |  | 1 |  |  |  |
| High school diploma or equivalent | 1.11 | (0.96–1.28) | 0.14 | 1.10 | (0.81–1.48) | 0.55 |  |
| Some college, undergraduate, graduate degree | 1.31 | (1.61–1.47) | <0.001 | 1.29 | (1.00–1.66) | 0.05 |  |
| **Type of professional activity** |  |  |  |  |  |  | <0.001 |
| Mostly sedentary, retired, other^c^ | 1.21 | (1.09–1.34) | <0.001 | 1.33 | (1.07–1.65) | 0.009 |  |
| Mostly active | 1 |  |  | 1 |  |  |  |
| **Prior divorce** |  |  |  |  |  |  | 0.15 |
| No | 1 |  |  | 1 |  |  |  |
| Yes | 1.07 | (0.99–1.16) | 0.10 | 0.93 | (0.77–1.12) | 0.45 |  |
| **Current household financial situation** |  |  |  |  |  |  | <0.001 |
| Comfortable, good | 1 |  |  | 1 |  |  |  |
| Barely making it, in debt | 1.17 | (1.07 –1.28) | <0.001 | 1.51 | (1.27–1.79) | <0.001 |  |
| **Self-perceived health status** |  |  |  |  |  |  | <0.001 |
| Very good, good | 1 |  |  | 1 |  |  |  |
| Acceptable | 1.32 | (1.22–1.43) | <0.001 | 1.71 | (1.44–2.03) | <0.001 |  |
| Poor or very poor | 1.89 | (1.63–2.18) | <0.001 | 3.21 | (2.49–4.14) | <0.001 |  |
| **Self-perceived dietary quality** |  |  |  |  |  |  | <0.001 |
| Excellent, very good | 1 |  |  | 1 |  |  |  |
| Good, acceptable | 1.59 | (1.47–1.72) | <0.001 | 2.21 | (1.83–2.68) | <0.001 |  |
| Poor | 5.00 | (3.92–6.37) | <0.001 | 14.38 | (10.11–20.45) | <0.001 |  |
| **BMI (kg/m^2^) category** |  |  |  |  |  |  | <0.001 |
| Underweight (<18.5) | 1.05 | (0.89–1.24) | 0.55 | 1.53 | (1.11–2.12) | 0.009 |  |
| Normal weight (18.5–24.9) | 1 |  |  | 1 |  |  |  |
| Overweight (25.0–29.9) | 1.31 | (1.21–1.43) | <0.001 | 1.55 | (1.29–1.87) | <0.001 |  |
| Obesity (≥30.0) | 1.87 | (1.68–2.07) | <0.001 | 3.02 | (2.46–3.70) | <0.001 |  |
| **Tobacco smoking status** |  |  |  |  |  |  | <0.001 |
| Never smoker | 1 |  |  | 1 |  |  |  |
| Former smoker | 1.36 | (1.26–1.47) | <0.001 | 1.31 | (1.09–1.57) | 0.004 |  |
| Current smoker | 3.08 | (2.76–3.44) | <0.001 | 6.07 | (5.00–7.37) | <0.001 |  |
| **Current e-cigarette use** |  |  |  |  |  |  | <0.001 |
| No | 1 |  |  | 1 |  |  |  |
| Yes | 1.41 | (1.17–1.69) | <0.001 | 1.53 | (1.14–2.06) | 0.004 |  |
| **Alcohol use, # glasses/week^d^** |  |  |  |  |  |  | <0.001 |
| 0 | 1 |  |  | 1 |  |  |  |
| < 2 | 0.93 | (0.84–1.04) | 0.19 | 0.97 | (0.75–1.26) | 0.84 |  |
| 2–6 | 1.60 | (1.43–1.79) | <0.001 | 2.98 | (2.30–3.86) | <0.001 |  |
| ≥ 7 | 19.19 | (16.24–22.67) | <0.001 | 54.17 | (39.77–73.78) | <0.001 |  |
| **Lack of affection during childhood** |  |  |  |  |  |  | <0.001 |
| No | 1 |  |  | 1 |  |  |  |
| Yes | 1.73 | (1.56–1.90) | <0.001 | 2.45 | (2.05–2.91) | <0.001 |  |
| **Self-reported lifetime prevalence or medication use for a mental disorder^e^** |  |  |  |  |  |  | <0.001 |
| No | 1 |  |  | 1 |  |  |  |
| Yes | 1.73 | (1.58–1.90) | <0.001 | 2.34 | (1.99–2.75) | <0.001 |  |
| BMI: Body Mass Index, CI: Confidence Interval, OR: Odds Ratio.  ^a^ Habit-forming risk behaviors include alcohol use disorders, nicotine dependence, food addiction, and Internet addiction; they were assessed by the Alcohol Use Disorders Identification Test (≥8 points), the 12-item Cigarette Dependence Scale (≥43 points), the modified Yale Food Addiction Scale 2.0, and the Internet Addiction Test (≥50 points), respectively.  ^b^ Values are obtained from a polytomous logistic regression model (reference=no risk behavior; n=27,036). Variables are mutually adjusted.  ^c^ Other=Without professional activity (homemaker, sick leave, unemployment, parental leave, disability) or not specified.  ^d^ 1 glass=10 g of ethanol.  ^e^ Mental disorders include memory impairment, Alzheimer’s disease, anorexia nervosa, anxiety disorders, bipolar disorder, depression, and insomnia. | | | | | | | |
